# Ceramide-Driven Mechanisms in Pulmonary Fibrosis

**DOI:** 10.3390/metabo16060421

**Published:** 2026-06-16

**Authors:** Zifan Li, Yaqian Li, Na Mao, Xuemin Gao, Hong Xu, Wenchen Cai, Tian Li

**Affiliations:** 1 School of Public Health, North China University of Science and Technology, Tangshan 063000, China; lizifan@stu.ncst.edu.cn (Z.L.);; 2 School of Traditional Chinese Medicine, North China University of Science and Technology, Tangshan 063000, China; 3 Health Science Center, North China University of Science and Technology, Tangshan 063000, China; 4 Hebei Key Laboratory of Rehabilitation Engineering and Regenerative Medicine, College of Nursing and Rehabilitation, North China University of Science and Technology, Tangshan 063000, China

**Keywords:** ceramide, pulmonary fibrosis, sphingolipid, acid sphingomyelinase, fibroblast activation

## Abstract

Pulmonary fibrosis, particularly idiopathic pulmonary fibrosis (IPF), is a chronic and progressive interstitial lung disease characterized by alveolar epithelial injury, fibroblast activation, and excessive extracellular matrix deposition, which collectively lead to respiratory failure. Despite the availability of antifibrotic agents, disease-modifying therapies remain limited. Emerging evidence has identified dysregulated sphingolipid metabolism, especially ceramide accumulation, as a key driver of fibrotic pathogenesis. Ceramide is a central bioactive lipid in the sphingolipid pathway that regulates multiple cellular processes, including apoptosis, inflammation, endothelial barrier dysfunction, and fibroblast activation, all of which contribute to pulmonary fibrosis. This review is a narrative review that systematically summarizes the biosynthetic and metabolic pathways of ceramide, with an emphasis on chain length-specific functions and the ceramide to S1P rheostat. We further discuss the mechanistic roles of ceramide in alveolar epithelial cell apoptosis, inflammatory responses, and vascular barrier disruption in fibrotic lung disease. Finally, we highlight emerging therapeutic strategies that target ceramide metabolism, including inhibitors of acid sphingomyelinase (ASMase) and serine palmitoyltransferase (SPT), and propose future directions for clinical translation.

## 1. Introduction

Pulmonary fibrosis, particularly idiopathic pulmonary fibrosis (IPF), is a chronic and progressive interstitial lung disease characterized by alveolar epithelial injury, fibroblast activation, and excessive extracellular matrix deposition, leading to respiratory failure and high mortality. Although antifibrotic agents such as pirfenidone and nintedanib can slow disease progression, no current therapy can reverse established fibrosis [1].

In recent years, bioactive lipids have emerged as critical regulators of pulmonary inflammation and fibrosis [2]. Among these, ceramide, a central hub in sphingolipid metabolism, has garnered particular attention. Ceramide is generated via three principal pathways: de novo synthesis by serine palmitoyltransferase (SPT), the sphingomyelinase pathway mediated by acid sphingomyelinase (ASMase), and the salvage pathway [3].

Accumulating evidence implicates ceramide dysregulation in the pathogenesis of pulmonary fibrosis. Elevated ceramide levels have been consistently observed in bleomycin-induced fibrosis models and in fibrotic lung diseases [4,5]. Ceramide upregulation causes pulmonary cell apoptosis and emphysema-like disease in mice. Mechanistically, ceramide promotes alveolar epithelial apoptosis, activates the NOD-like receptor thermal protein domain associated protein 3 (NLRP3) inflammasome, disrupts endothelial barrier function, and facilitates fibroblast activation [6,7]. Notably, the balance between ceramide and sphingosine-1-phosphate (S1P), termed the sphingolipid rheostat, critically influences cell fate, and its disruption has been documented in fibrotic lungs [8,9].

As a narrative review, we searched PubMed, Web of Science, and Google Scholar up to March 2026 using combinations of the following keywords: “ceramide”, “sphingolipid”, “pulmonary fibrosis”. “idiopathic pulmonary fibrosis”, “silicosis”, “acid sphingomyelinase”, “ceramide synthase”, “S1P”, “fibroblast”, “macrophage”, “alveolar epitheial cells”, “endothelial cells”, “inflammation”. Inclusion criteria: peer-reviewed original articles and reviews written in English, directly related to ceramide metabolism in lung fibrosis or related lung diseases. Exclusion criteria: non-English articles, conference abstracts without full text, and studies unrelated to lung biology. Key findings from these articles are synthesized in this review.

This review summarizes ceramide metabolism, its pathophysiological roles in pulmonary fibrosis, and emerging therapeutic strategies that target this signaling pathway.

## 2. Ceramide Metabolism and Signaling Regulation

### 2.1. Biosynthetic Pathways of Ceramide

**Figure 1:**
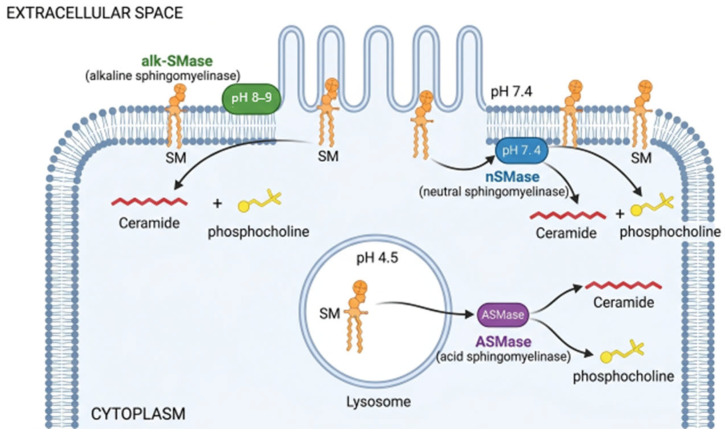
Schematic representation of sphingomyelin degradation pathways in the human lung Sphingomyelin (SM) in the plasma membrane is hydrolyzed by three sphingomyelinases (SMases) to generate ceramide and phosphocholine. Acid sphingomyelinase (ASMase) functions within lysosomes at acidic pH (∼4.5), neutral sphingomyelinase (nSMase) acts on the cytoplasmic leaflet of the plasma membrane at neutral pH, and alkaline sphingomyelinase (alk-SMase) is localized on the extracellular surface or microvilli at alkaline pH (∼8–9). Ceramide produced by these pathways serves as a central signaling lipid in pulmonary fibrosis. Abbreviations: ASMase, acid sphingomyelinase; nSMase, neutral sphingomyelinase; alk-SMase, alkaline sphingomyelinase; SM, sphingomyelin.

Ceramide is generated within cells through three principal pathways: the de novo synthesis pathway, the sphingomyelinase pathway, and the salvage pathway [4] (Figure 1). De novo synthesis begins in the endoplasmic reticulum with the condensation of serine and palmitoyl coenzyme A, a reaction catalyzed by serine palmitoyltransferase (SPT). This reaction generates 3 ketodihydrosphingosine, which is subsequently reduced by 3 ketodihydrosphingosine reductase (KDSR) and N-acylated by ceramide synthase (CERS) to produce dihydroceramide. Finally, dihydroceramide is desaturated by dihydroceramide desaturase (DEGS) to generate ceramide. Accordingly, targeted knockout of key de novo synthesis enzymes can significantly reduce total ceramide levels and delay disease progression [7,10,11].

SPTLC1 is essential for myeloid differentiation during hematopoiesis, and its deficiency disrupts sphingolipid biosynthesis, leading to endoplasmic reticulum stress that impairs myelopoiesis while sparing erythropoiesis [12]. In addition, specific mutations identified in SPTLC1 cause rare genetic disorders, including hereditary sensory neuropathy type 1 (HSN1A) and juvenile amyotrophic lateral sclerosis (ALS) [13,14]. As the initiating and rate-limiting enzyme in sphingolipid biosynthesis, inhibition of SPTLC1 or SPTLC2 with myriocin ameliorates ceramide-dependent lung pathologies by restoring metabolic and autophagic homeostasis. These pathologies include cigarette smoke-induced metabolic dysfunction, neutrophil elastase-driven airway inflammation, radiation-induced pulmonary fibrosis, and fungal infection in cystic fibrosis [15,16,17,18]. Moreover, inhibition of SPTLC1 significantly suppresses lipopolysaccharide (LPS) induced pulmonary microvascular endothelial cell (PMVEC) apoptosis and the resulting apoptosis-dependent delayed barrier dysfunction in the lung [19].

Sphingomyelin is hydrolyzed by sphingomyelinase (SMase) to generate ceramide and phosphocholine. Based on their optimal pH, SMases are classified into acid sphingomyelinase (ASMase, also known as sphingomyelin phosphodiesterase 1, SMPD1), neutral sphingomyelinase (nSMase, also known as SMPD2, SMPD3, and SMPD4), and alkaline sphingomyelinase (alk-SMase, also known as ectonucleotide pyrophosphatase phosphodiesterase 7, ENPP7). Among these, ASMase is rapidly activated under conditions of inflammation, oxidative stress, and infection, serving as a key enzyme for rapid ceramide generation. In cystic fibrosis, inhalation of various ASMase inhibitors normalizes ceramide levels in the lungs, thereby reducing inflammation and infection without systemic side effects [20]. In a murine model of asthma, ASMase deficiency or inhibition reduces T helper 2 (TH2) cytokine production from T cells, protecting against allergic airway inflammation and hyperresponsiveness [21]. Furthermore, in pulmonary fibrosis-related diseases such as chronic obstructive pulmonary disease (COPD), emphysema, and silicosis, ASMase mediates fibrosis by promoting ceramide accumulation via oxidative stress and inflammation, whereas its inhibition reduces fibrosis and restores extracellular matrix homeostasis [22,23]. Even partial deletion of Smpd1 leads to reduced C16 to C24 ceramide ratios and increased de novo sphingolipid synthesis and S1P levels. These changes collectively inhibit stress-induced lung endothelial cell apoptosis while promoting exaggerated inflammation and post-injury cell proliferation with altered autophagy, thereby limiting lung vascular injury and enhancing repair following inflammatory insults [24]. Collectively, these findings establish ASMase as a central regulator of ceramide-mediated pathophysiology across multiple chronic lung diseases, highlighting its potential as a broad-spectrum therapeutic target.

The salvage pathway primarily operates in acidic compartments such as late endosomes and lysosomes. There, ceramide metabolites—particularly sphingosine generated from ceramide degradation—are reacylated by ceramide synthases to regenerate ceramide, forming a metabolic cycle. In IPF, sphingosine kinase 1 (SPHK1) promotes fibrogenesis, whereas sphingosine-1-phosphate lyase (S1PL) plays a protective role, suggesting that targeting SphK1 offers a potential therapeutic approach [25]. Targeting the nuclear SPHK2 to S1P signaling axis, which epigenetically regulates proinflammatory gene expression and impairs cystic fibrosis transmembrane conductance regulator (CFTR) activity, may represent a promising therapeutic strategy for inflammatory lung disorders such as pneumonia, cystic fibrosis, and cigarette smoke-induced COPD [26,27].

### 2.2. The Ceramide/S1P Rheostat

Ceramide and S1P exert opposing biological functions. Ceramide promotes cell cycle arrest, apoptosis, and inflammation, whereas S1P promotes cell survival, proliferation, and migration. Their dynamic balance, known as the sphingolipid rheostat, is critical for cell fate and tissue homeostasis [28]. This rheostat concept has since evolved to incorporate greater complexity, including S1P inside-out signaling, acyl chain-specific ceramide functions, and compartment-specific metabolite production [8]. In the lungs, disruption of the proapoptotic (ceramide) and prosurvival (S1P) balance contributes to pathological remodeling. Specifically, elevated ceramide levels have been linked to epithelial cell apoptosis and endothelial barrier dysfunction, which are key events in the initiation and progression of pulmonary fibrosis [4].

Disruption of ceramide-to-S1P metabolism is an important determinant of the emphysema phenotype in COPD [29]. This disruption is characterized by decoupled ceramide and S1P levels and reduced SPHK1 activity in the distal lung. Sphingosine-1-phosphate receptors (S1PRs) play distinct, cell-specific roles in pulmonary physiology and disease. S1PR1 is highly expressed in lung endothelial cells and is downregulated in IPF. Endothelial-specific *S1pr1* deletion promotes inflammation and fibrosis, whereas S1PR1 activation with IMMH002 preserves endothelial barrier integrity and alleviates bleomycin-induced pulmonary fibrosis [9]. Genetic or pharmacological inhibition of S1PR2 enhances macrophage-derived interleukin 33 (IL-33) release, thereby attenuating sepsis-induced lung injury [30]. S1PR3 is upregulated in alveolar epithelial cells of IPF patients and fibrotic mice. Epithelial-specific deletion or pharmacological inhibition of S1PR3 attenuates fibrosis, reduces inflammation and collagen deposition, and improves alveolar capillary barrier integrity by enhancing tight junction proteins [31].

### 2.3. Chain Length-Specific Functions of Ceramide

The acyl chain length of ceramide, ranging from C14 to C26, is determined by the specific acyl coenzyme A utilized during synthesis, and ceramides of different chain lengths exhibit distinct biological functions. The ceramide synthase (CerS) family comprises six members (CerS1 to CerS6), each preferentially synthesizing ceramides with specific chain lengths. For example, CerS5 and CerS6 primarily generate C16 ceramide, whereas CerS2 predominantly catalyzes the synthesis of very long chain ceramides, specifically C22 to C24 [3].

Recent studies have suggested that specific ceramide subspecies regulate cellular homeostasis, although much of this evidence comes from non-lung tissues and requires validation in the pulmonary context. CERS2 deficiency has been reported to impair insulin secretion in pancreatic islet cells [32]; palmitate activates CERS2 to promote very long chain ceramide synthesis in peripheral tissues [33]; and CERS5 has been implicated in aortic valve stenosis [34]. However, direct evidence linking individual CerS isoforms to pulmonary fibrosis in humans remains limited.

Emerging evidence indicates that specific ceramide subspecies play roles in pulmonary fibrosis, although most studies are cross-sectional and small in scale. In fibrotic lung disease, total ceramide levels are elevated, but the correlation with HRCT scores differs between stable and progressive disease [35]. This suggests that ceramide profiles may change with disease activity, but causality cannot be inferred.

In cystic fibrosis (CF), long-chain ceramides (C18, C20) and the very long-chain C24:1 are elevated, while sphingosine is reduced [36]. However, saturated very long-chain ceramides are downregulated in CF cells and mice [37]. This species-specific divergence highlights the need to distinguish between saturated and monounsaturated very long-chain ceramides. Fenretinide restores the balance by downregulating Cers5, decreasing long-chain and increasing saturated very long-chain ceramides [37]. Of note, ref. [36] studied CFTR modulator therapy in plasma, whereas ref. [37] examined fenretinide in preclinical models and a small trial—direct comparison requires caution.

In silicosis patients (*n* = 50), C16, C18, C20, and C24 ceramides are elevated, but only weakly correlated with clinical parameters; longitudinal studies are needed [38]. In bleomycin-induced fibrosis, CerS2-deficient mice show compensatory C16 elevation and airway inflammation [39]. However, these mice also have liver and brain pathology, so the lung phenotype may reflect systemic effects. In COVID-19 autopsy lungs, C16 is elevated ninefold with reversed C16/C24 ratios [40]; whether this occurs in non-fatal cases or is a cause vs. consequence remains unknown. Diesel exhaust particles increase C1 and C2 in macrophages [41], but relevance to IPF (not primarily caused by diesel exhaust) requires further investigation.

Although C16 is consistently proapoptotic and proinflammatory in multiple models, chain-length-dependent effects are context-dependent. In CerS2-null mice, loss of C24 (protective) accompanies C16 elevation, making it unclear which drives pathology [39]. In silicosis, multiple species are elevated, challenging a simple “C16-bad, C24-good” binary model [38]. In CF, CFTR modulators reduce long-chain and increase very-long-chain C24 ceramide [36], consistent with fenretinide effects [37], supporting opposing functions of different chain lengths. However, most human data come from small studies (e.g., only 25 patients in the ELX/TEZ/IVA arm of [36]) and lack prospective validation. Thus, ceramide pathogenicity cannot be generalized without considering cell type, disease stage, and metabolic context. A summary of key studies linking specific ceramide subspecies to pulmonary fibrosis and related lung diseases is provided in Table 1.

## 3. Role of Ceramide in the Pathogenesis of Pulmonary Fibrosis

### 3.1. Promotion of Inflammatory Responses

Recent studies have uncovered that ceramide functions as a core messenger driving macrophage inflammatory responses, forming a vicious cycle that promotes disease progression. Specifically, ceramide can bind to cysteinyl leukotriene receptor 2 (CYSLTR2) and pyrimidinergic receptor P2Y6 (P2RY6), activating downstream Gq protein and subsequently the NLRP3 inflammasome [45]. Ceramide can also interact with formyl peptide receptor 2 (FPR2), activating Gi signaling to reduce cyclic adenosine monophosphate (cAMP) levels [46]. In addition, ceramide mediates voltage-dependent anion channel 1 (VDAC1) oligomerization, leading to mitochondrial DNA leakage into the cytosol, which activates the cyclic GMP-AMP synthase (cGAS) to stimulator of interferon gene (STING) signaling pathway [7,47]. Furthermore, in the absence of IL-10 signaling, toll-like receptor 2 (TLR2) activated macrophages synthesize saturated very long chain ceramides via CERS2 that maintain persistent nuclear factor kappa B (NF-κB) family member REL activity, driving chronic inflammatory gene expression [48].

Inflammatory stimuli potently upregulate ceramide generation [49], which in turn amplifies inflammatory responses, establishing a vicious cycle that sustains pathological inflammation in the lung [50]. Ceramide overproduction activates the Txnip to NLRP3 inflammasome axis in alveolar macrophages, leading to early IL-1β release followed by endothelial IL-6 and tumor necrosis factor alpha (TNF-α) secretion. Collectively, these events drive pulmonary microvascular endothelial barrier dysfunction and acute lung injury [6]. Ceramide and its metabolites play complex and sometimes opposing roles in inflammation, with ceramide promoting proinflammatory processes and apoptosis, S1P supporting cell growth and survival, and ceramide 1-phosphate (C1P) exhibiting both proinflammatory and cell type or tissue-specific anti-inflammatory effects [51].

Importantly, recent evidence has established extracellular vesicles (EVs) as critical carriers that transport bioactive ceramides between pulmonary cell populations, particularly from macrophages to endothelial cells, thereby propagating a paracrine ceramide-mediated injury signal across the lung microenvironment [44]. Diesel exhaust particle exposure drives de novo ceramide biosynthesis in macrophages, which in turn impairs mitochondrial respiration, elevates hydrogen peroxide (H_2_O_2_) production, and increases systemic inflammation [41]. Conversely, mitochondrial dysfunction has been shown to be both a cause and a consequence of ceramide accumulation. Ceramide can directly bind to and inhibit mitochondrial electron transport chain complexes, particularly complex I and III, leading to increased mitochondrial reactive oxygen species (mtROS) production. This mtROS burst further promotes the enzymatic activity of ASMase and de novo ceramide synthesis, creating a feed-forward vicious cycle that amplifies cellular injury and inflammatory signaling. Thus, this reciprocal regulation positions mitochondria both as a target and an amplifier of ceramide-driven pathology in fibrotic lungs.

While the above studies demonstrate ceramide-driven inflammatory signaling in animal and cell models, direct evidence in human IPF lungs remains limited. The majority of mechanistic data come from mouse models of acute lung injury (e.g., LPS), silicosis, or COPD—not from IPF. Only one study has directly examined ceramide-induced NLRP3 activation in human IPF samples [6], and it was limited to a small number of explanted lungs. Furthermore, whether the ceramide-inflammation vicious cycle operates in the same manner in human IPF as in acute models is unknown. Notably, anti-inflammatory therapies have largely failed in IPF clinical trials, raising the question of whether targeting ceramide-driven inflammation alone would be sufficient. Thus, while ceramide is a promising pro-inflammatory mediator in preclinical models, its role in human IPF pathogenesis requires further validation, ideally through longitudinal sampling and correlation with disease progression.

### 3.2. Induction of Alveolar Epithelial Cell Apoptosis

Repeated injury and aberrant apoptosis of alveolar epithelial cells are considered critical initiating events in pulmonary fibrosis. Ceramide is a well-established proapoptotic signaling molecule that can induce alveolar epithelial cell apoptosis through multiple mechanisms [5]. In pulmonary alveolar proteinosis, ceramide and other sphingolipids are massively accumulated, with more than a 130-fold increase, particularly the long-chain species d18:1/20:0 and d18:1/24:0, contributing to a proapoptotic alveolar environment [42]. C16 ceramide generated by SMPD1 activation upon mechanical ventilation or cyclic stretch promotes autophagy-mediated alveolar epithelial cell death by inducing LC3B II formation and LC3B to FAS receptor interaction, leading to extrinsic apoptosis [52]. Ceramide induces alveolar epithelial cell apoptosis via mutual upregulation with RTP801, a hypoxia and oxidative stress sensor, creating a self-amplifying loop that drives lung injury [53]. In allergic asthma, allergen-induced elevation of lung ceramide levels drives airway epithelial apoptosis, reactive oxygen species (ROS) generation, and neutrophilic inflammation independent of oxidative stress, suggesting that ceramide is a key trigger of severe asthmatic pathology [45]. Ceramide (C2) also disrupts the alveolar epithelial barrier by downregulating tight junction proteins, thereby increasing permeability and potentially contributing to noncardiogenic lung edema in acute lung injury [54].

Most evidence for ceramide-induced alveolar epithelial apoptosis derives from mouse models (bleomycin, mechanical ventilation, allergen challenge) or from transformed cell lines. Human data are sparse: one study reported ceramide accumulation in alveolar proteinosis patients [50], but this is a rare disease distinct from IPF. In IPF, direct evidence of ceramide-driven epithelial apoptosis in patient lung tissue is lacking. Moreover, the functional relevance of ceramide-induced apoptosis versus other pro-apoptotic pathways (e.g., FAS/FASL, p53) in IPF has not been dissected. It is also controversial whether epithelial apoptosis in IPF is a driver of fibrosis or a secondary response to injury. Therefore, while ceramide is a pro-apoptotic signal in experimental systems, its causal role in human IPF epithelial cell death remains to be established.

Several pharmacological agents have been shown to interrupt the crosstalk between ceramide and inflammation in preclinical models of pulmonary fibrosis and related lung diseases. (1) ASMase inhibitors (desipramine, amitriptyline) reduce ceramide accumulation and subsequent NLRP3 inflammasome activation, decreasing IL-1β and IL-6 release in silicosis and COPD models [23]. (2) Myriocin, an SPT inhibitor, blocks de novo ceramide synthesis and attenuates radiation-induced pulmonary fibrosis by reducing proinflammatory cytokine production [17]. (3) Fenretinide, which modulates CerS5 activity, restores the balance between long-chain and very long-chain ceramides and reduces inflammation in cystic fibrosis [37]; its effects in IPF have not been tested. (4) N-acetylcysteine (NAC) can suppress ASMase activation and subsequent ceramide generation, partly through the Nrf2/HO-1 pathway, as shown in silicosis [23]. (5) S1PR3 inhibitors (e.g., TY52156) not only preserve barrier function but also reduce macrophage-driven inflammation in bleomycin-induced fibrosis [31]. However, none of these agents have been specifically evaluated in IPF patients for their ability to disrupt ceramide-driven inflammation, representing a key translational gap.

### 3.3. Disruption of Alveolar Endothelial Barrier Integrity

In LPS-induced acute lung injury models, ASMase knockout mice exhibit less severe endothelial injury and pulmonary edema. These findings further confirm the critical role of ceramide in endothelial barrier disruption. In emphysema, cigarette smoke reduces lung glucosylceramide (GlcCer) via suppression of glucosylceramide synthase (GCS), leading to increased lysosomal pH, impaired autophagic flux, inhibition of mammalian target of rapamycin (mTOR) signaling, and subsequent lung endothelial cell apoptosis [55]. The lung homing peptide CGSPGWVRC directly binds to C16 ceramide on lung vascular endothelium, inducing ceramide-rich platform formation and acid sphingomyelinase activation without triggering apoptosis, and this lipid-based targeting system can be applied for pulmonary imaging and lung immunization against COVID-19 [56]. In COPD, macrophage-derived ceramides transported via extracellular vesicles drive disease pathogenesis by inducing endothelial cell dysfunction [49].

While the studies cited above demonstrate that ceramide can disrupt endothelial barrier function and induce endothelial apoptosis in various experimental systems, several important limitations and controversies should be noted. First, most of these studies were performed in cultured human pulmonary microvascular endothelial cells or in rodent models of acute injury (LPS, mechanical ventilation, or cigarette smoke). Whether similar mechanisms operate in the chronically fibrotic lung of IPF patients remains unknown, as endothelial injury in IPF is likely more indolent and may differ mechanistically from acute stress-induced ceramide generation. Second, the specific ceramide species responsible for endothelial dysfunction in IPF have not been identified—most studies use non-physiologic short-chain ceramides (e.g., C2-ceramide) or rely on non-selective enzyme inhibitors, which may have off-target effects. Third, the only human data linking ceramide to lung endothelial injury come from COVID-19 autopsy studies [40], which showed C16 accumulation and ceramide staining in vascular endothelium. However, COVID-19 ARDS is a distinct disease from IPF, and it is unclear whether similar ceramide-driven endothelial pathology occurs in IPF. Fourth, the role of ceramide in endothelial cells may be context-dependent: some studies report that ceramide promotes endothelial apoptosis [5], whereas others suggest that very long-chain ceramides may be protective. Thus, while ceramide is a plausible mediator of endothelial injury, direct evidence in IPF patients is lacking, and the species-specific and context-dependent effects require further investigation.

### 3.4. Regulation of Fibroblast Activation and Collagen Deposition

Fibroblasts are the primary effector cells responsible for extracellular matrix production in pulmonary fibrosis, and their activation into proliferative, matrix-secreting myofibroblasts represents a critical step in fibrotic progression. Emerging evidence indicates that ceramide metabolism and the ceramide to S1P rheostat play important roles in regulating fibroblast fate and function.

The balance between ceramide and S1P appears to be a key determinant of fibroblast survival versus apoptosis. Ceramide generally promotes fibroblast apoptosis, whereas S1P supports fibroblast proliferation, migration, and resistance to cell death [28]. In IPF lungs, dysregulation of this balance favors S1P-mediated prosurvival signaling. SPHK1, the enzyme that generates S1P, is upregulated in fibrotic lung tissues, and targeting SPHK1 reduces fibrogenesis in preclinical models [25].

S1PRs also contribute to fibroblast activation in a cell-specific manner. S1PR3 is upregulated in fibroblasts from IPF patients, and pharmacological inhibition or genetic deletion of S1PR3 attenuates bleomycin-induced pulmonary fibrosis and reduces collagen deposition [31]. Additionally, CerS2-deficient mice, which exhibit elevated C16 ceramide and reduced very long chain ceramides, show increased susceptibility to fibrotic stimuli [39].

Although direct evidence in IPF fibroblasts is lacking, studies in other cell types suggest several pathways through which ceramide could regulate fibroblast fate: (i) activation of protein phosphatase 2A (PP2A) leading to AKT dephosphorylation and apoptosis [57]; (ii) direct binding and activation of cathepsin D, a lysosomal protease [58]; (iii) complex cross-talk with TGF-β/Smad signaling—ceramide can either inhibit (C2 ceramide [59]) or promote TGF-β responses depending on species and context; (iv) activation of JNK/p38 stress kinases implicated in myofibroblast differentiation [60]. None of these have been validated in human IPF fibroblasts, and literature reports contradictory effects that likely reflect differences in ceramide species, concentration, and cell context. Systematic studies using primary IPF fibroblasts are needed to resolve these gaps.

Compared with the well-established roles of ceramide in epithelial apoptosis, macrophage inflammation, and endothelial barrier disruption, the direct evidence for ceramide regulation of lung fibroblast activation in IPF is the weakest. Most studies on fibroblasts come from systemic sclerosis, cancer-associated fibroblasts, or non-lung tissues. In pulmonary fibrosis, the majority of evidence is indirect: from CerS2 knockout mice [39], from S1PR3 inhibition [31], or from correlation studies. Direct treatment of primary human IPF fibroblasts with specific ceramide species (e.g., C16:0) has been reported in only a few studies, often with contradictory results—some show pro-fibrotic effects, others pro-apoptotic effects depending on concentration and cell passage number. Moreover, the balance between ceramide and S1P in fibroblasts may be cell-autonomous or influenced by paracrine signals from other lung cells. Therefore, the precise role of ceramide in fibroblast-to-myofibroblast differentiation in IPF remains an important knowledge gap that requires further mechanistic investigation.

**Figure 2:**
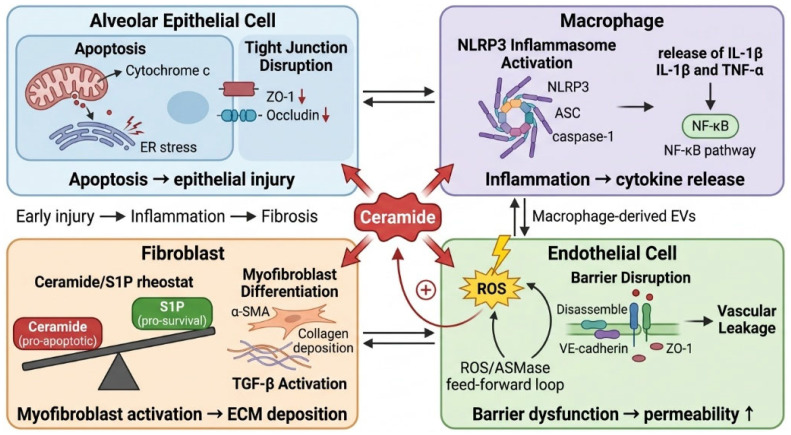
Pathophysiological pathways of ceramide in pulmonary fibrosis.

The roles of ceramide on various cell types during pulmonary fibrosis are shown in Figure 2.

Ceramide (central hub) drives pathogenic effects in four key lung cell types. In alveolar epithelial cells, ceramide induces apoptosis (mitochondrial pathway, ER stress) and disrupts tight junctions (ZO-1, occludin). In macrophages, ceramide activates the NLRP3 inflammasome, leading to IL-1β and TNF-α release, which amplifies inflammation via NF-κB. In endothelial cells, ceramide causes barrier dysfunction (disassembly of VE-cadherin and ZO-1) and promotes vascular leakage; mitochondrial ROS production establishes a feed-forward loop with ASMase. In fibroblasts, the ceramide/S1P rheostat is tilted toward S1P, promoting myofibroblast differentiation (α-SMA, collagen) and extracellular matrix deposition. Bidirectional arrows indicate cellular crosstalk, for example, macrophage-derived extracellular vesicles delivering ceramides to endothelial cells. The bottom progression arrows illustrate disease stages from early injury through inflammation to fibrosis. Abbreviations: α-SMA, alpha-smooth muscle actin; ASC, apoptosis-associated speck-like protein; ECM, extracellular matrix; ER, endoplasmic reticulum; IL, interleukin; NLRP3, NLR family pyrin domain containing 3; NF-κB, nuclear factor kappa B; ROS, reactive oxygen species; S1P, sphingosine-1-phosphate; TNF-α, tumor necrosis factor alpha; ZO-1, zonula occludens-1.

## 4. Challenges and Future Perspectives

Despite significant progress, several important challenges remain. The chain length-specific functions of ceramide species require further investigation, as in many studies, C16 ceramide appears predominantly proapoptotic and proinflammatory, whereas very long chain ceramides such as C22 to C24 may exert opposing effects. However, as discussed in Section 2.3, this binary model is an oversimplification: in silicosis patients, multiple ceramide species (C16, C18, C20, C24) are all elevated [38], and in CerS2-null mice, the loss of C24 ceramide accompanies compensatory C16 elevation, making it difficult to attribute pathology to C16 alone [39]. Furthermore, emerging evidence from other fields suggests that the biological functions of ceramide are not only determined by its acyl chain length but also by its subcellular localization. Ceramide-enriched domains on the mitochondrial outer membrane, endoplasmic reticulum, and plasma membrane have been shown to exert distinct effects on apoptosis, autophagy, and inflammatory signaling. However, the subcellular distribution of specific ceramide species in pulmonary fibrosis remains largely unknown. Future studies are needed to elucidate how compartment-specific ceramide pools contribute to cell-type-specific pathological outcomes in the fibrotic lung.

The cell-type-specific roles of ceramide signaling in the lung also remain incompletely understood. Ceramide metabolism in epithelial cells, macrophages, endothelial cells, and fibroblasts likely produces divergent functional outcomes that could be dissected using emerging techniques such as single-cell metabolomics and conditional knockout models. Most current evidence, however, comes from rodent models or immortalized cell lines; human data on cell-type-specific ceramide signaling in IPF are almost nonexistent. Although pharmacological inhibitors of ASMase and SPT have demonstrated efficacy in preclinical models, clinical trials in patients with IPF are currently lacking. Inhalation-based delivery systems may offer advantages by achieving high local drug concentrations while minimizing systemic side effects. Notably, inhaled ASMase inhibitors (e.g., amitriptyline) have been tested in cystic fibrosis patients, showing normalization of pulmonary ceramide levels without systemic toxicity [20]. However, similar formulations have not yet been developed or tested for IPF.

The potential utility of plasma ceramide species as disease biomarkers warrants further validation. The ratio of C16 to C24 ceramide, in particular, may serve as a prognostic or predictive biomarker in fibrotic lung disease. However, as noted in Section 2.3, the direction of change for very long-chain ceramides varies across diseases, and the C16/C24 ratio has not been consistently validated in IPF-specific cohorts. Large-scale prospective clinical studies are needed to confirm these associations. Furthermore, existing human studies are predominantly cross-sectional with small sample sizes (typically *n* < 50) and do not control for potential confounding by antifibrotic medications (pirfenidone, nintedanib), which themselves may alter ceramide levels.

Several obstacles impede the translation of ceramide-targeting therapies to IPF patients. First, species differences in ceramide metabolism exist: mice and humans have different expression patterns and substrate specificities of ceramide synthases (CerS) and sphingomyelinases. For example, the potency and selectivity of inhibitors (e.g., myriocin for SPT) may differ between species. Second, animal models of IPF, particularly the bleomycin model, do not fully recapitulate the human disease: fibrosis is acute and self-limiting, whereas IPF is chronic and progressive. Third, human biomarker studies have been limited to small, single-center cohorts with heterogeneous disease stages and medication use; no ceramide-based biomarker has been prospectively validated in IPF. Fourth, drug delivery to the lung interstitium, where fibrosis occurs, may require inhalation formulations, which have not yet been developed for ASMase or SPT inhibitors. Overcoming these challenges will require parallel preclinical validation in human ex vivo lung tissue and carefully designed early-phase clinical trials with biomarker endpoints.

Several unresolved controversies remain. First, while C16 ceramide is widely considered “pathogenic”, the *CesS2* knockout mouse—which has massively elevated C16—develops inflammation and emphysema but not fibrosis [39]. This suggests that high C16 alone may be insufficient to drive fibrosis; additional factors (e.g., TGF-β, extracellular matrix remodeling) are required. Second, the cell-type specificity of ceramide action is poorly understood: ceramide may be pro-apoptotic in epithelial cells but pro-survival or pro-fibrotic in fibroblasts, depending on the metabolic context. Third, the contribution of dietary sphingolipids versus endogenous synthesis to lung ceramide pools in IPF is unknown. Future studies should address these controversies using conditional knockout mice, single-cell lipidomics, and carefully designed longitudinal human cohort studies.

Currently, two drugs, pirfenidone and nintedanib, are approved for IPF treatment [1,61]. To date, no study has directly demonstrated that either agent lowers ceramide levels in lung tissue or specifically targets the ceramide/S1P rheostat. Nevertheless, indirect mechanistic links exist. Pirfenidone is known to inhibit TGF-β signaling and reduce pro-inflammatory cytokine production [62,63]; given that TGF-β can upregulate sphingosine kinase 1 (SphK1) expression and S1P generation [25]. Pirfenidone may secondarily modulate ceramide-related pathways. Nintedanib, a tyrosine kinase inhibitor, has been shown to alter sphingolipid profiles in experimental fibrosis models [64] and to affect plasmalogen (pPE) lipid species in IPF patients, with such changes correlating with treatment response [65]. Importantly, neither drug has been systematically evaluated for its effect on specific ceramide subspecies or the ceramide/S1P rheostat [25]. Direct head-to-head comparisons between ceramide-targeting agents (e.g., ASMase or SPT inhibitors) and these approved drugs in preclinical models or clinical trials are currently lacking. Such comparisons would be valuable to determine whether targeting ceramide offers additional or synergistic benefits over existing therapies. A summary of emerging therapeutic strategies targeting ceramide metabolism and their current status is presented in Table 2.

## 5. Conclusions

Ceramide, as a central hub in sphingolipid metabolism, participates in the pathogenesis and progression of pulmonary fibrosis through multiple mechanisms, including the induction of cell apoptosis, promotion of inflammation, disruption of endothelial barriers, and activation of fibroblasts. Targeting ceramide signaling pathways, particularly inhibiting ASMase or SPT activity and restoring the ceramide to S1P balance, holds promise as a novel therapeutic strategy for pulmonary fibrosis. However, given the current predominance of preclinical evidence and the lack of validated human biomarkers, careful translation through well-designed clinical studies is essential before ceramide-targeting therapies can be recommended for IPF. With a deepening understanding of the ceramide regulatory network, precision interventions based on sphingolipid metabolism may bring new hope to patients with pulmonary fibrosis.

## Figures and Tables

**Table 1:** Key preclinical and clinical evidence linking ceramide subspecies to lung diseases.

Author, Year	Model System	Ceramide Species	Key Findings
Petrache, 2005 [5]	Mouse (C16 ceramide)	C16	Ceramide upregulation causes alveolar epithelial apoptosis and emphysema-like disease
Petrache, 2013 [39]	CerS2 knockout mouse	C16 (compensatory)	CerS2 deficiency leads to spontaneous airway inflammation and enhanced fibrotic susceptibility
Griese, 2019 [42]	Pulmonary alveolar proteinosis patients	d18:1/20:0, d18:1/24:0	>130-fold ceramide accumulation in alveolar space
James, 2021 [43]	Allergic asthma mouse model	Total ceramide	Allergen-induced ceramide elevation drives epithelial apoptosis and neutrophilic inflammation
Ouyang, 2023 [6]	LPS-induced ALI mouse model	Ceramide	Ceramide activates Txnip/NLRP3 axis, leading to endothelial barrier dysfunction
Petrache, 2023 [40]	COVID-19 patients	C16:0, C24:0	C16:0 ceramide elevated 9-fold in lung; reversed C16/C24 ratio
Aksu Kaplan, 2026 [38]	Silicosis patients	C16, C18, C20, C24	Multiple ceramide species elevated in plasma
Huang, 2025 [44]	COPD mouse model	Long-chain, very long-chain	Macrophage-derived extracellular vesicles deliver ceramides to endothelial cells

**Table 2:** Therapeutic strategies targeting ceramide metabolism in pulmonary fibrosis.

Target	Agent	Preclinical Efficacy	Clinical Status
ASMase	Desipramine, Amitriptyline	Reduces fibrosis in silicosis and COPD models [23]	Not evaluated in IPF; approved for depression (off-label use not tested)
SPT	Myriocin	Ameliorates radiation-induced pulmonary fibrosis [17]	Preclinical only; toxicity concerns limit translation
S1PR1	IMMH002	Preserves endothelial barrier, alleviates bleomycin-induced fibrosis [9]	Preclinical only; phase I for psoriasis (not IPF)
S1PR3	Pharmacological inhibitor	Attenuates fibrosis, enhances tight junctions [31]	Preclinical only; no clinical trial in IPF
SPHK1	SPHK1 inhibitor	Reduces fibrogenesis [25]	Preclinical only; no IPF trial
CerS5	Fenretinide	Restores long-chain/very long-chain balance [37]	Phase II in cystic fibrosis (NCT03265288); not evaluated in IPF
Multiple	Pirfenidone/Nintedanib (approved antifibrotics)	Shown to partially reduce ceramide levels in animal models (indirect effect) [65,66]	Approved for IPF (not ceramide-targeting)

## Data Availability

No new data were created or analyzed in this study.

## Abbreviations

The following abbreviations are used in this manuscript:

α-SMA	Alpha-smooth muscle actin
ALI	Acute lung injury
ASMase	Acid sphingomyelinase
BAL	Bronchoalveolar lavage
C1P	Ceramide 1-phosphate
CerS	Ceramide synthase
CF	Cystic fibrosis
CFTR	Cystic fibrosis transmembrane conductance regulator
COPD	Chronic obstructive pulmonary disease
COVID-19	Coronavirus disease 2019
DEGS	Dihydroceramide desaturase
DHS1P	Dihydrosphingosine-1-phosphate
ECM	Extracellular matrix
EMT	Epithelial–mesenchymal transition
EV	Extracellular vesicle
HRCT	High-resolution computed tomography
IPF	Idiopathic pulmonary fibrosis
KDSR	3-ketodihydrosphingosine reductase
LPS	Lipopolysaccharide
mtROS	Mitochondrial reactive oxygen species
NAC	N-acetylcysteine
NF-κB	Nuclear factor kappa B
NLRP3	NLR family pyrin domain containing 3
nSMase	Neutral sphingomyelinase
PMVEC	Pulmonary microvascular endothelial cell
ROS	Reactive oxygen species
S1P	Sphingosine-1-phosphate
S1PL	S1P lyase
S1PR	S1P receptor
SphK	Sphingosine kinase
SPT	Serine palmitoyltransferase
TGF-β	Transforming growth factor-beta
TLR	Toll-like receptor
TNF-α	Tumor necrosis factor-alpha
